# Development of Automated 3D LiDAR System for Dimensional Quality Inspection of Prefabricated Concrete Elements

**DOI:** 10.3390/s24237486

**Published:** 2024-11-24

**Authors:** Shuangping Li, Bin Zhang, Junxing Zheng, Dong Wang, Zuqiang Liu

**Affiliations:** 1 Changjiang Spatial Information Technology Engineering Co., Ltd., Wuhan 430010, China; lishuangping@cjwsjy.com.cn (S.L.); zhangbin@cjwsjy.com.cn (B.Z.); liuzuqiang@cjwsjy.com.cn (Z.L.); 2 School of Civil and Hydraulic Engineering, Huazhong University of Science and Technology, Wuhan 430074, China

**Keywords:** prefabricated concrete element, dimensional quality inspection, 3D LiDAR system, point cloud processing

## Abstract

The dimensional quality inspection of prefabricated concrete (PC) elements is crucial for ensuring overall assembly quality and enhancing on-site construction efficiency. However, current practices remain heavily reliant on manual inspection, which results in high operator dependency and low efficiency. Existing Light Detection and Ranging (LiDAR)-based methods also require skilled professionals for scanning and subsequent point cloud processing, thereby presenting technical challenges. This study developed a 3D LiDAR system for the automatic identification and measurement of the dimensional quality of PC elements. The system consists of (1) a hardware system integrated with camera and LiDAR components to acquire 3D point cloud data and (2) a user-friendly graphical user interface (GUI) software system incorporating a series of algorithms for automated point cloud processing using PyQt5. Field experiments comparing the system’s measurements with manual measurements on prefabricated bridge columns demonstrated that the system’s average measurement error was approximately 5 mm. The developed system can provide a quick, accurate, and automated inspection tool for dimensional quality assessment of PC elements, thereby enhancing on-site construction efficiency.

## 1. Introduction

Compared with the manufacturing industry, the architecture, engineering, and construction (AEC) industry has been in a state of labor intensity [1], under digitization [2], and low efficiency [3] in the past few decades. Currently, construction industrialization (CI) [4] has attracted the extensive attention of a large number of researchers and engineers to improve the present situation of AEC. CI refers to industrialized production through prefabricated construction, modular construction, and assembled installation to replace the decentralized, low-level, and inefficient handicraft production methods in the traditional construction industry [4]. Prefabricated concrete (PC) elements, a key product of CI, can improve efficiency and save 70% of construction time and 43% of labor costs [5,6]. In general, PC elements are assembled to form a complete structure [7]. However, it is noteworthy that linkages, such as rebars, connecting PC elements are frequently prone to elevated stress concentrations, making them the most susceptible component in the entire structural system [5,8]. Thus, dimension quality inspection of PC elements, especially the rebars at the connecting parts, is indispensable for quality control and subsequent efficient assembly on-site [9].

Currently, the dimensional quality inspection of PC elements primarily depends on manual methods involving tape measures. This approach is time-consuming and heavily dependent on the skill of the operator. To address the issue, non-contact inspection methods, including Light Detection and Ranging (LiDAR) and imaging techniques [5], have been developed for geometric quality inspection, which has improved detection efficiency and accuracy to a certain extent. For instance, Yuan et al. [10] utilized a cost-effective RGB-D sensor to accurately detect the arrangement of reinforcement and concrete protective layers. This approach facilitated the rapid identification of discrepancies in reinforcement placement, significantly enhancing detection efficiency with millimeter-level accuracy. Tommaselli and Reiss [11] achieved non-contact plane size calculation by utilizing a camera and a laser rangefinder based on a two-dimensional image-based approach. Ye et al. [12] presented a bridge deflection measurement method utilizing machine vision and digital image processing technology, enabling real-time, non-contact, and cost-effective bridge deflection measurement. Although the aforementioned approaches for measuring deformations and dimensions of bridges are commendable in their efficiency and cost-effectiveness, they are susceptible to the impact of external environmental factors, with particular emphasis on the illumination condition [5]. The reliance on illumination conditions undoubtedly diminishes the dependability of their precision.

To mitigate the effects of external environmental factors and acquire more precise geometric dimension data of PC elements, laser scanners can be utilized to capture comprehensive visual information, primarily including spatial coordinate information pertaining to the PC elements. Similar to camera-based technology, the performance of laser scanners is also affected by various factors, such as environmental conditions (e.g., lighting, dust, surface reflectivity) and the characteristics of the scanned objects (e.g., shape, surface color, roughness) [13,14]. Nonetheless, because of the capability of laser scanners to accomplish non-contact, fast, and high-precision measurement, LiDAR technique has been extensively employed in the construction industry [15], such as geometric quality inspection [5,9,16,17,18], 3D model reconstruction [19,20,21], schedule monitoring [22,23,24,25], and safety management [26,27]. Notably, significant advancements have been achieved in the research on the quality inspection of connection parts in PC components, particularly quality inspection related to rebars. This encompasses rebar point cloud classification, rebar spacing and length evaluation [17,28,29], rebar diameter classification [30,31], rebar layout standardization [16,32], etc. For example, Zhao et al. [28] proposed a one-class classifier, a clustering algorithm, and an alpha-shape algorithm to automatically recognize and measure the rebars, concrete, and sleeves of PC elements. Li et al. [31] presented a scan planning approach to determine the optimal scan position of a laser scanner for accurate rebar diameter prediction, thus achieving the inspection of rebar diameter prior to concrete pouring. Existing research predominantly utilizes commercial LiDAR equipment, requiring the involvement of skilled personnel for object scanning and subsequent point cloud processing, which imposes substantial technical limitations. Furthermore, the high cost associated with commercial LiDAR systems increases the financial burden for small and medium-sized enterprises [9]. Hence, it is essential to improve the automation level of LiDAR-based quality detection methods while simultaneously reducing the expenses associated with the LiDAR-based inspection method.

To address the aforementioned issues, this study developed a 3D LiDAR system to automatically identify and measure the dimensional quality of PC elements. Specifically, the hardware system combined camera and LiDAR components and was developed to capture 3D point cloud and image data. A graphical user interface (GUI) software system integrated with a series of algorithms for automated point cloud processing was developed using PyQt5 (version number: 5.15). The effectiveness and precision of the developed method were verified through field experiments comparing the results with manual measurements. The primary objective of this study is to improve the dimensional quality inspection process for PC elements, particularly bridge columns, through automated 3D LiDAR scanning. The key advantage of this system lies in its ability to provide quick, accurate, and repeatable measurements, thereby reducing human error and operator dependency in the inspection process.

## 2. The Proposed 3D LiDAR System

**Figure 1:**
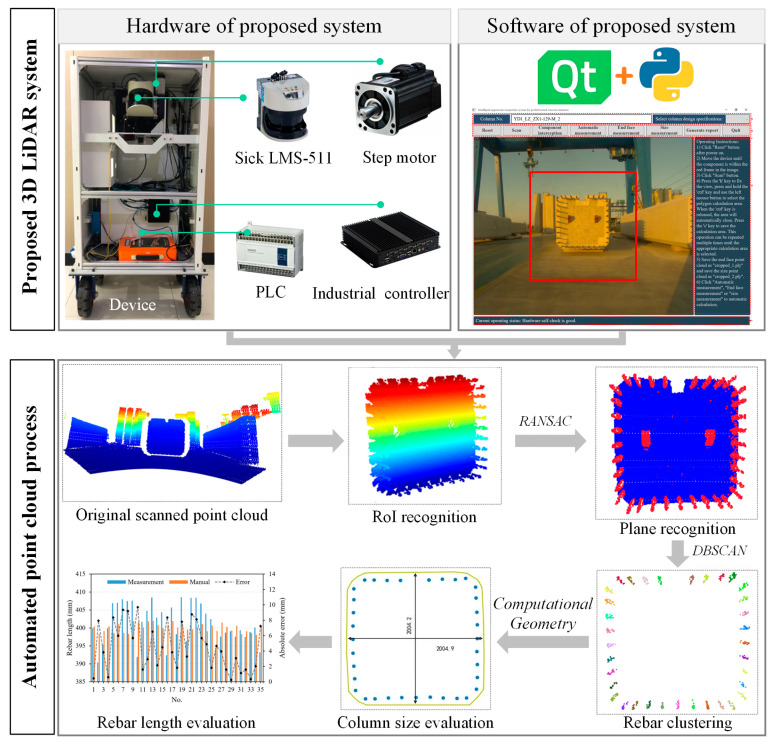
Research framework.

The overall framework of the system developed in this study is illustrated in Figure 1. The system consists of both hardware and software components. The hardware primarily includes a 2D linear LiDAR, a stepper motor, a PLC, and an industrial computer. The software, developed using Python, is implemented based on the third-party library named PyQt. The system is employed to scan prefabricated columns and capture their 3D point clouds. A series of point cloud processing tasks are performed using machine learning algorithms, ultimately enabling the measurement of the rebars and geometric dimensions of the column end face. Additionally, the system automatically generates a measurement report.

### 2.1. Hardware Composition of Proposed System

#### 2.1.1. Image Acquisition of the Column End Face

The mainstream camera types in the market mainly include complementary metal oxide semiconductor (CMOS) cameras and charge-coupled device (CCD) cameras. When the CCD photosensitive device is working, it accepts the optical signal, converts it into a charge signal through the photosensitive area array, and generates the driving signal by the driving circuit for point and transmission, thereby realizing the pixel matrix shooting. Compared with CMOS cameras, CCD sensors generally have a higher signal-to-noise ratio and better image quality in low-light conditions, which is particularly important for capturing detailed column images in on-site construction environments with varying light conditions. This feature helps to achieve clearer, more accurate images of the column and rebar surfaces, especially when performing the column quality inspection at night. While CMOS sensors are often cheaper and more widely adopted by industrial camera manufacturers, we have chosen a CCD camera for our system to capture high-resolution, high-quality images for accurate column measurements in daylight and low-light conditions.

#### 2.1.2. Selection of LiDAR Scanner Device

LiDAR scanners found in the prevailing market can be categorized into two distinct types: triangular ranging (TT) LiDAR and time-of-flight (TOF) LiDAR, each following a distinct technical approach. The operational mechanism of TT LiDAR entails the emission of laser light that illuminates the target object, while a linear CCD captures the resulting reflected light. Due to the separation between the laser and the detector, objects situated at varying distances manifest in diverse locations within the CCD. By leveraging the principles of trigonometry, the distance to the target object can be ascertained. Conversely, TOF LiDAR involves the emission of laser pulses, whose departure and return times are precisely timed using a dedicated timer. The timer records the duration taken for the light to travel and return. By subtracting the departure and return times, the “flight time” of the light is deduced. Exploiting the constant speed of light, the distance can be readily calculated once the speed and time variables are known [33].

Considering factors such as the size of the inspection object, application scenarios, and the characteristics of the two LiDARs, we have chosen TOF LiDAR to acquire the point cloud data. This decision is based on the following reasons [34]: (1) Measuring distance: TT LiDAR struggles to distinguish objects at longer distances due to diminishing position differences on the CCD. As a result, it may not meet the requirements for measuring the distance to significant components such as prefabricated bridge columns. In contrast, TOF LiDAR employs pulsed laser sampling, enabling measurements at longer distances and mitigating the impact of ambient light. (2) Accuracy: trigonometry exhibits high accuracy at close range. However, as the distance increases, its measurement accuracy deteriorates. In contrast, TOF LiDAR relies on flight time, and its time measurement accuracy remains relatively stable regardless of distance. Considering the diameter of the rebar is around 40 mm, and the length of the rebar is around 400 mm, a resolution of 5 mm is required for spatial and depth measurement. Thus, the stepper motor is configured to rotate by 0.05° in response to a positive signal, resulting in a measurement accuracy of approximately 5 mm. The TOF LiDAR used in the system achieves these resolutions by using a combination of advanced processing algorithms and hardware capabilities, which were designed to meet these requirements while maintaining high efficiency. (3) Speed: TT LiDAR typically has a maximum speed below 20 Hz, while TOF LiDAR can achieve approximately 30–50 Hz. A high rotation speed is valuable for point cloud imaging, particularly for the rapid movement of the 2D LiDAR relative to the column during the scanning process in this work. Thus, we chose TOF LiDAR because its high sampling rate enables us to collect a dense point cloud efficiently, reducing the time required for data acquisition.

#### 2.1.3. Overall Structure of Proposed Hardware System

**Figure 2:**
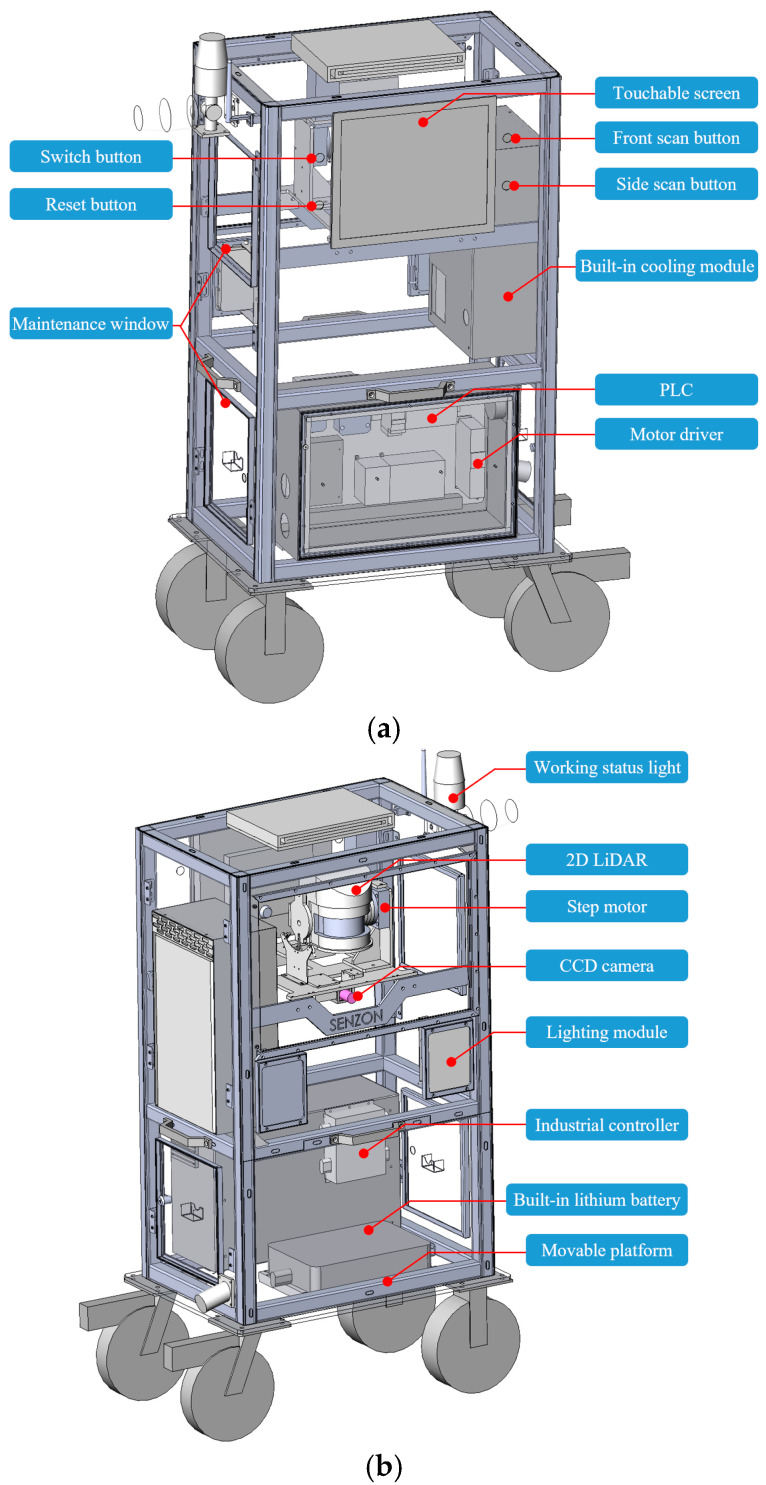
The developed device: (**a**) the operating end of the device and (**b**) internal structure of the device.

Given the inherent precision and sensitivity of the laser scanner, stringent considerations must be made regarding its working environment. Meticulous attention should be paid to avoiding direct exposure to sunlight and the erosive effects of rain. Therefore, it is imperative to establish the LiDAR system within a controlled and enclosed space that effectively mitigates the direct influence of external conditions. These measures are paramount for ensuring the sustained long-term functionality and reliability of the LiDAR apparatus. Considering the operational requirements of the TOF LiDAR, which involves the emission of laser pulses and the reception of corresponding reflected light signals, it is essential to ensure the LiDAR’s working surface with the open space. This critical step safeguards the integrity and accuracy of the emitted and received light, preventing undesirable interference or distortion in the data acquisition process. To facilitate the acquisition of point cloud data, the deployment of a wheeled platform trolley proves advantageous, enabling the LiDAR system to be conveniently positioned as needed. According to the above design concepts, the comprehensive design schematics of the device are presented in Figure 2a,b.

The proposed hardware system encompasses various components, such as 2D LiDAR, CCD camera, industrial controller, programmable logic controller (PLC), stepper motor, power supply, etc. The stepper motor plays a pivotal role in driving the pitch direction movement of the 2D LiDAR, facilitating the acquisition of 3D point cloud data. In our system, a stepper motor and driver are utilized to control the pitch rotation of the 2D LiDAR, with the stepper motor and driver working together to form a precise closed-loop control system that guarantees accurate positioning. The stepper motor, driver, and PLC were all sourced from the same manufacturer (www.xinje.com (accessed on 20 November 2024)) to ensure optimal compatibility and seamless communication between the system’s components. The decision to choose 2D LiDAR instead of directly employing 3D LiDAR is primarily driven by the need to achieve an optimal balance between cost-effectiveness and measurement accuracy. Additionally, this choice enables more seamless integration of the LiDAR components within our developed system architecture. To ensure precise control, the stepper motor is meticulously regulated by the PLC (www.xinje.com (accessed on 20 November 2024)). This PLC is selected for its robust performance in industrial environments and its easy integration with the stepper motor driver and other system components. Serving as the control center of the entire hardware system, the industrial controller seamlessly integrates the multimodal data obtained from the PLC, 2D LiDAR, and CCD camera, thereby enabling cooperative control of the overall hardware structure. The main specifications of the industrial controller utilized in this study are as follows: an Intel i7-9700 processor, 16 GB of RAM, an NVIDIA GTX 1050 graphics card, and the Windows 10 operating system. The hardware system operates on a lithium battery, while different components are interconnected with the overall device through hardware conversion tools. Taking into account the equipment’s erecting specifications and the selected drive method, the TOF LiDAR system is ultimately installed within a cabinet-type enclosure mounted on a wheeled trolley base. The open space surrounds the LiDAR work surface, facilitating unimpeded scanning operations. The LiDAR can be reset and scanned through the simplified four-button interface. After the scanning is completed, subsequent geometric quality assessments are performed using the developed GUI, as elaborated in Section 2.2.2. An illumination module is also incorporated to facilitate column quality inspection tasks during nighttime. The innovative hardware system enables automated inspection of prefabricated components without the need for skilled professional control.

### 2.2. Algorithm Design and GUI Implementation

A point cloud is a dataset of points in a particular coordinate system. These points contain rich information, including 3D coordinates, color, classification value, intensity value, acquisition time, etc. As a considerable collection of three-dimensional coordinate points, point cloud data have the following characteristics: huge amount of data, discrete nature, and radioactive distribution. Therefore, different methods are needed for point cloud processing and analysis.

#### 2.2.1. Point Cloud Preprocessing and Automated Evaluation

Point cloud processing in this paper mainly involves point cloud visualization, downsampling, target plane recognition, target plane reconstruction, and point cloud segmentation. The different point cloud processing procedures are as follows:

1. Point cloud visualization. Point cloud data consist of a vast collection of three-dimensional coordinate points. To enable computer reading and calculations, the data are converted into the “.ply” standard format for storage. For point cloud visualization, the Open3D library in Python is utilized.

2. Point cloud downsampling. During the preprocessing of point clouds, it is common to encounter noise and handle the massive amount of 3D point cloud data. Various preprocessing techniques, such as denoising, downsampling, and point cloud structuring, are employed to enhance processing efficiency and improve data quality. Among these techniques, the voxel downsampling algorithm [35] is a commonly used technique and offers advantages, such as high computational efficiency and uniform distribution of sample points. Thus, we employ the voxel downsampling algorithm to simplify the point cloud.

3. Target plane recognition. After preprocessing and downsampling the point cloud, we obtain the reduced point cloud. The plane recognition technique is employed to separate the end face of the column, where the rebars are embedded. This division provides convenience for subsequent calculations. In this work, the Random Sample Consensus algorithm (RANSAC) [36] is utilized to identify the plane within the point cloud. It achieves this by iteratively estimating the parameters of a mathematical model from a set of observational datasets, which may contain outliers. Outliers can result from extreme noise values, incorrect measurement methods, or erroneous assumptions about the data. The RANSAC algorithm is considered an indeterminate algorithm, meaning it has a probability of yielding a reasonable result and more reasonable handling of outliers. Besides, increasing the number of iterations improves the likelihood of obtaining an accurate plane recognition. The recognition result includes the plane equation as (a, b, c, d), where the coordinates (*x*, *y*, *z*) of each point on the plane satisfy the equation a*x* + b*y* + c*z* + d = 0.

4. Target plane reconstruction. After the column end face is segmented, the plane is reconstructed to facilitate the subsequent calculation of geometric dimensions. We employ the convex hull algorithm [37] to envelop each plane’s boundary point cloud. In a two-dimensional Euclidean space, the convex hull can be visualized as a rubber band that encloses all the points. Given a set of points on a two-dimensional plane, the convex hull is a convex polygon formed by connecting the outermost points, effectively encompassing all the points in the set. The fundamental concept of the convex hull algorithm is based on the notion that two points determine a line. If all the remaining points lie on the same side of the line, then these two points are part of the convex hull. Otherwise, they are not. The algorithm consists of two steps: (1) Pairing all the points in the point set to create *n ×* (*n* − 1)/2 straight lines. (2) For each line, rechecking whether the remaining (*n* − 2) points lie on the same side of the line. To determine the relative position of a point *p*_3_(*x*_3_, *y*_3_) with respect to the line *p*_1_*p*_2_ (coordinates: *p*_1_(*x*_1_, *y*_1_) and *p*_2_(*x*_2_, *y*_2_)), Equation (1) can be utilized to determine if point *p*_3_ is on the left or right side of the line *p*_1_*p*_2_. A positive result indicates that *p*_3_ is on the left side, while a negative result indicates the opposite.

(1) $$\left|\begin{array}{ccc} x_{1} & y_{1} & 1\\ x_{2} & y_{2} & 1\\ x_{3} & y_{3} & 1 \end{array}\right|=x_{1}y_{2}+x_{3}y_{1}+x_{2}y_{3}-x_{3}y_{2}-x_{2}y_{1}-x_{1}y_{3}$$

5. Point cloud segmentation. After identifying the column end face, Density-Based Spatial Clustering of Applications with Noise (DBSCAN) [38] is a density-based spatial clustering algorithm that identifies clusters of arbitrary shapes in a spatial database with noise. Unlike the k-means clustering algorithm [39], DBSCAN is capable of handling various cluster shapes. The DBSCAN algorithm has two parameters: ε, which defines the neighborhood radius for points within the cluster, and Minpoints, which determines the minimum number of points required to form a cluster. The algorithm can effectively group closely packed points and identify isolated points as noise. Considering the specific appearance characteristics of the column end face and rebar point cloud, this work utilizes the DBSCAN algorithm for point cloud processing. By utilizing the DBSCAN algorithm, we aim to effectively segment the rebar point cloud and filter out noisy data.

**Figure 3:**
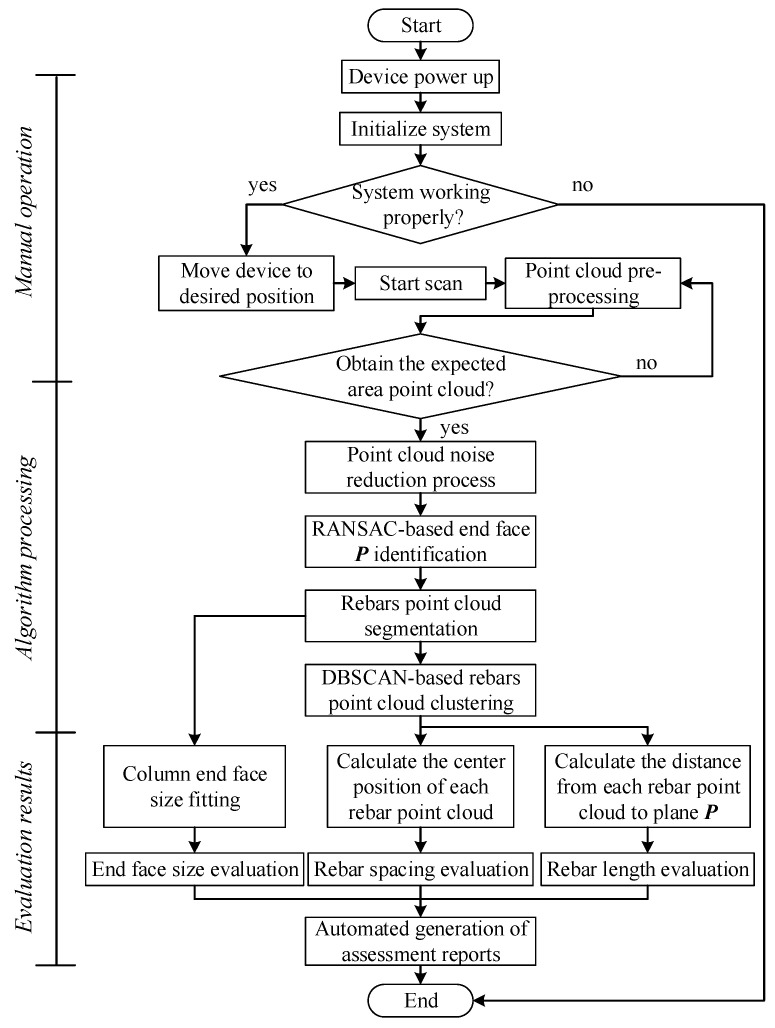
Workflow of column end face automatic inspection system.

Finally, the point cloud data of each rebar are projected onto the column end face, and the RANSAC-based circle fitting method is employed to obtain the center point coordinates of each rebar. Subsequently, the determination of the rebar spacing can be accurately achieved through the computation of the Euclidean distance between the coordinates of the central points of adjacent rebars. Besides, to determine the length of the *i*th rebar, the projection distance *d_i_*_,*j*_ from the *j*th point *P_i_*_,*j*_ of the rebar to the plane ***P*** could be calculated. Then, the 2*σ* rule of normal distribution as a tolerance value was employed to estimate the rebar length. This statistical method helps to mitigate the impact of outliers and noise in the point cloud data, ensuring a more reliable calculation of the length. The overall procedure for automatic inspection of the column end face is illustrated in Figure 3.

#### 2.2.2. GUI for Proposed System

**Figure 4:**
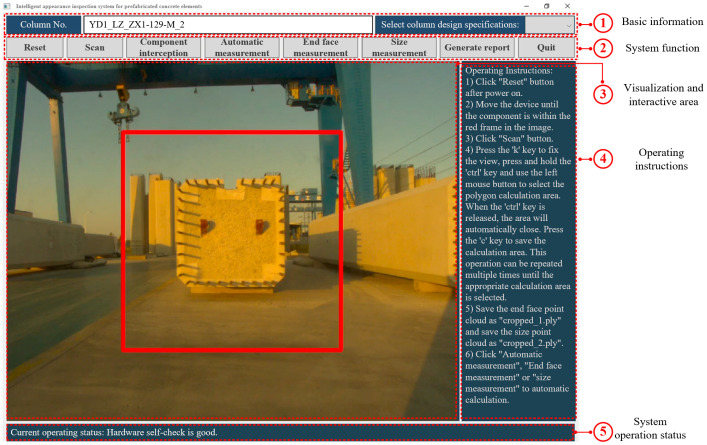
Software system of the proposed device.

To make it easier to interact with hardware devices and geometric quality assessment, a user-friendly GUI based on PyQt5 [40] was developed using the Python language, and the system interface is shown in Figure 4. PyQt5 is highly popular among developers and is regarded as a preeminent Python binding for the Qt C++ framework, which is a cross-platform framework.

The proposed GUI involves five parts, which are basic information, system function, visualization and interactive area, operating instructions, and system operation status. In the basic information section, the system allows the user to input essential information regarding the column components, which facilitates the storage of data for distinguishing different as-produced columns. For the system functional section, the user has control over LiDAR scanning, point cloud processing, automatic calculation of column end face dimensions, rebar spacing and length, and the generation of reports. These functions form the core of the GUI and incorporate the algorithms discussed in Section 3.1. The GUI includes a visualization and interactive area to assist the user in positioning the device correctly for scanning. Additionally, it provides an interactive area for point cloud processing and the presentation of evaluation results. Clear operating instructions are provided to guide the user through the GUI’s operation, while the system operation status displays logs, alerts, and other relevant information.

## 3. Field Experiment and Verification

### 3.1. Field Experiment

**Figure 5:**
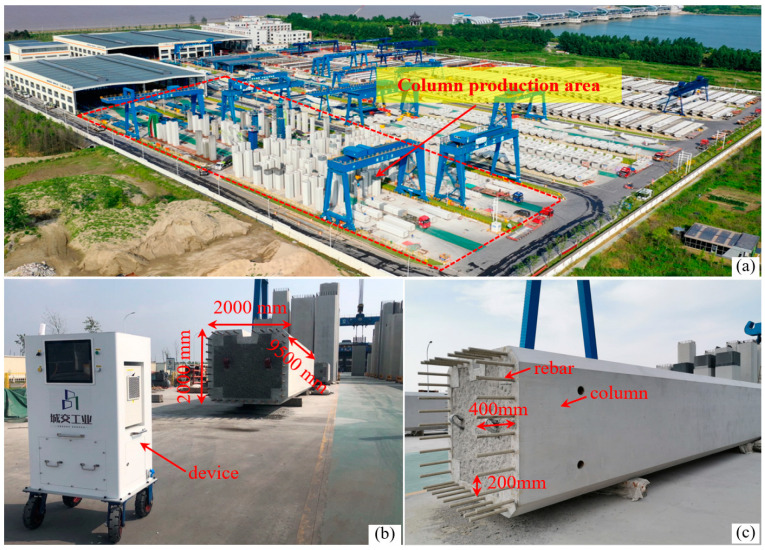
Experiment in the field: (**a**) column production area, (**b**) column scanning based on the proposed system, and (**c**) basic information of bridge column.

The examined case study pertains to an industrialized manufacturing facility located in eastern China, serving as a prominent example of large-scale production of prefabricated elements specifically designed for bridge columns, as illustrated in Figure 5a. The assessment of production quality for a selected column is conducted employing the developed column inspection equipment, as illustrated in Figure 5b. Figure 5c provides essential information regarding the fundamental dimensions of the column. The dimensions of the end face of the column are 2000 mm in length and 2000 mm in width. The top section of the column end face encompasses 35 pre-embedded rebars. The designed spacing between the rebars is approximately 200 mm, with an elongation length of 400 mm.

**Figure 6:**
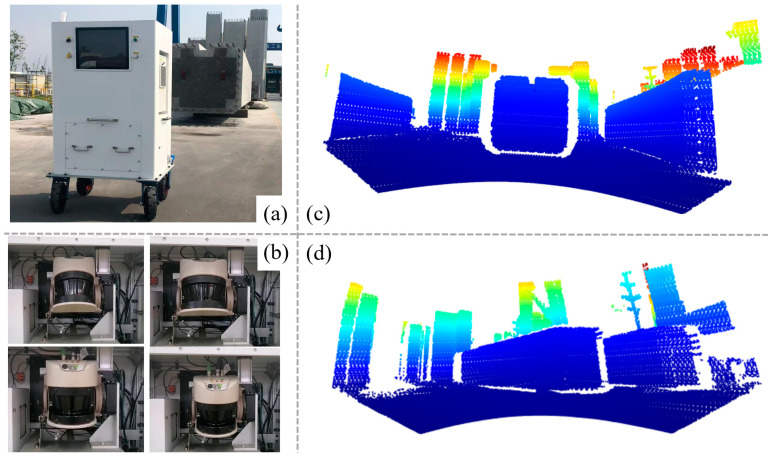
Point cloud visualization of the column: (**a**) position of frontal scan, (**b**) rotation process of 2D LiDAR, (**c**) results from frontal scan, and (**d**) results from side-scan.

The prefabricated bridge columns were scanned at two distinct locations using our proposed 3D scanner. For the scanning process, our proposed device was strategically positioned approximately 5 m from the column end face (Figure 6a) and about 6 m away from the side of the column. This setup allowed for two independent scans to capture detailed point cloud data from both the top and side views of the column. The suitable location determined using the image guide box is indicated by the red rectangle in Figure 4. This positioning typically took only 1 to 2 min. During the scanning process, the 2D LiDAR systematically rotated in a vertical plane from the upper position to the lower position, as illustrated in Figure 6b. The point cloud visualization of the end face scene and the side scene of the prefabricated bridge column are provided in Figure 6c and Figure 6d, respectively. Before initiating the point cloud data analysis, a manual segmentation process was applied to the raw point cloud, ensuring that all extraneous elements in the scene, apart from the column itself, were identified and removed. This process took about 1 minute. Furthermore, the processing and analysis of the point cloud data were conducted using the PyCharm-integrated development environment, with all coding and computational tasks performed in the Python programming language. This automatic analysis of the point cloud took approximately 1 minute. During this process, the RANSAC algorithm required about 0.24 s to identify planes, while the DBSCAN algorithm clustered the rebar point cloud in less than 0.5 s.

### 3.2. Experimental Analysis and Results

**Figure 7:**
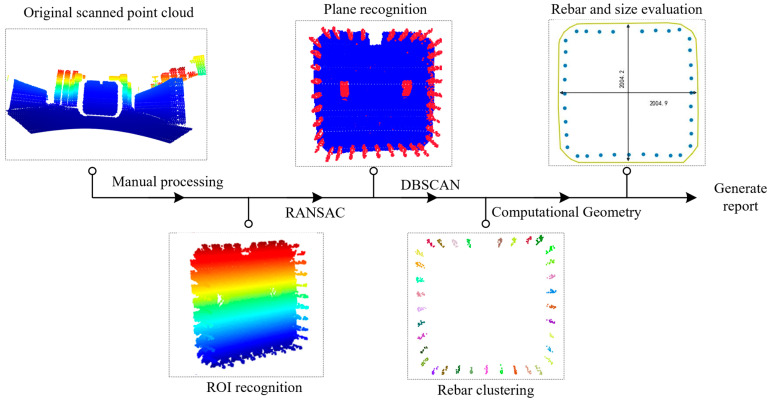
Point cloud processing and assessment of PC column.

Upon acquiring the initial point cloud data, the pivotal outcomes derived from the point cloud analysis are shown in Figure 7. The point cloud analysis encompasses various crucial stages, namely point cloud preprocessing, identification of the column end face, point cloud clustering, evaluation of rebar and end face dimensions, and the subsequent generation of an inspection report. Through the approach in Section 3.1, the RANSAC algorithm is employed to extract the plane of the column end face, facilitating the derivation of length and width dimensions using the convex hull algorithm. Subsequently, segmenting the entire point cloud yields individual point clouds for each rebar using the DBSCAN clustering method. By calculating the Euclidean distance between the point cloud projection onto the end face and the distances among adjacent rebars, both rebar length and spacing can be determined. An electronic evaluation report is produced based on the aforementioned calculation results. Ultimately, the automated assessment of column production quality is achieved.

In terms of performance, the software can process point cloud data in real-time with an average processing time of approximately 2 minutes per scan for the initial data acquisition, including point cloud cleaning and segmentation. The software handles high-density point clouds efficiently, thanks to the optimization of the algorithms and the computational power of the industrial computer. Besides, the software runs seamlessly with the hardware components, with communication between the PLC, stepper motor, and LiDAR being managed in real-time to ensure precise data collection and analysis.

**Figure 8:**
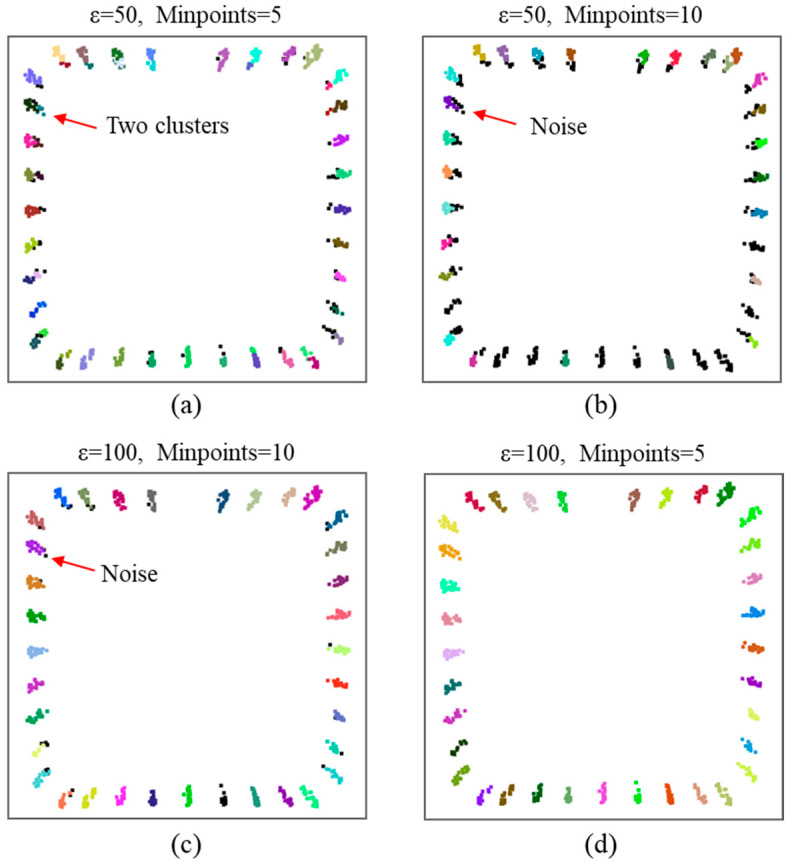
Rebars clustering results under different parameters: (**a**) ε = 50, Minpoints = 5; (**b**) ε = 50, Minpoints = 10; (**c**) ε = 100, Minpoints = 10; (**d**) ε = 100, Minpoints = 5.

In the context of point cloud clustering, accurate segmentation of rebars is essential for determining their spacing and length. Figure 8 presents the clustering results derived from various parameter configurations of the DBSCAN algorithm, revealing cluster counts of 52, 27, 34, and 35 for Figure 8a through Figure 8d, respectively. Notably, the parameter settings illustrated in Figure 8d produce satisfactory clustering results. The parameters ε and Minpoints are set to 100 and 5, respectively. In contrast, Figure 8a shows that a single rebar is incorrectly categorized into two separate clusters due to excessively small parameter values, leading to a significant deviation from the actual scenario. While increasing the Minpoints parameter enhances the accuracy of the clustering results, it simultaneously introduces substantial noise. Conversely, an increase in the ε value effectively reduces noise interference. Therefore, the judicious selection of clustering parameters is vital for achieving reliable outcomes, as evidenced by the sensitivity of the ε value in influencing the accuracy of the clustering results in Figure 8d.

**Figure 9:**
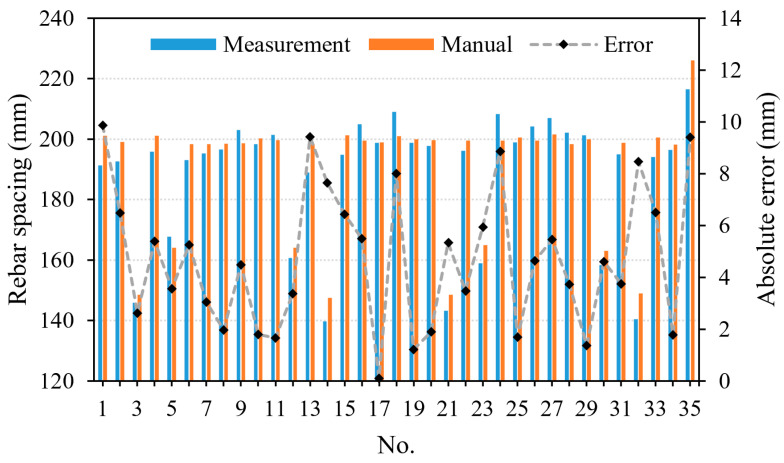
Absolute difference values of embedded rebar spacing.

**Figure 10:**
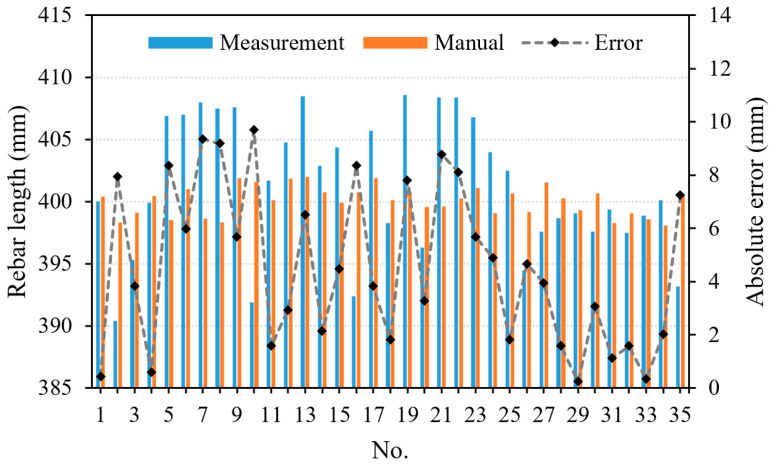
Absolute difference values of embedded rebar length.

The detecting results of the column end face are 2004.2 (height) × 2004.9 mm (width). Compared with the proposed system with manual measurement results, the errors in height and width directions are 4.2 mm and 4.9 mm, respectively. The average error of the size of the column end face is 4.55 mm. The detection of rebar spacing exhibits a maximum discrepancy of 9.4 mm (illustrated in Figure 9), with an average discrepancy of 4.71 mm. The detection of the embedded rebar length indicates a maximum discrepancy of 9.7 mm (illustrated in Figure 10), with an average discrepancy of 4.54 mm. All of the aforementioned discrepancies satisfy the allowable error requirements for the design specifications [41,42], where the allowable tolerances for rebar spacing and length are required to be within 10 mm and 20 mm, respectively. The proposed system can be employed to swiftly and precisely assess the dimensional characteristics of prefabricated bridge columns on-site, replacing the conventional manual detection approach. As a result, it enhances the efficiency of on-site construction for prefabricated bridges and reduces costs associated with the inspection process.

## 4. Conclusions

In the present study, a 3D LiDAR-based automatic recognition and dimension measuring device and algorithm were developed for quality inspection of PC bridge elements. Comparison experiments were conducted in the field to verify the accuracy and effectiveness of the proposed system. The main conclusions are summarized as follows:

(1) Hardware Development: A hardware system that integrates the main components, such as a CCD camera, LiDAR, and step motor driver, was developed, which realizes the acquisition of a 3D point cloud from a 2D LiDAR scanner.
(2) Algorithm Development: A series of algorithms for point cloud processing were developed, including noise reduction, reconstruction, segmentation, and clustering. Besides, a user-friendly GUI software system integrated with the above algorithms was developed using PyQt5 for portable applications.
(3) Accuracy Verification: The accuracy of the developed system was verified in a field experiment compared with manual measurements. The maximum differences observed for size, spacing, and length were 4.9 mm, 9.4 mm, and 9.7 mm, respectively, while the average errors were 4.55 mm, 4.71 mm, and 4.54 mm, respectively, which verified the effectiveness and practicality of the proposed method.

Future work can be conducted to address some aspects of the issues. For instance, integrating multimodal information from camera and LiDAR data allows for the automatic extraction of end-face point cloud information, enabling end-to-end fully automated point cloud processing without human intervention. This significantly enhances inspection efficiency. Additionally, the integration of deep learning-based point cloud processing methods into the proposed system has the potential to enhance evaluation accuracy, enabling high-standard quality inspection on construction sites. Furthermore, it is important to test the system with different PC elements or scales to ensure its versatility and reliability in on-site scenarios. Also, a detailed comparative study between our system and a commercial LiDAR system could further validate the reliability and accuracy of our system under various conditions.

## Data Availability

Data will be made available on request.
